# Brain Abscess: A Rare Clinical Case with Oral Etiology

**DOI:** 10.1155/2022/5140259

**Published:** 2022-01-04

**Authors:** André João da Silva Pais Rocha Pereira, Ana Teresa Tavares, Marcelo Prates, Natacha Ribeiro, Luís Filipe Fonseca, Maria do Rosário Marques, Francisco Proença

**Affiliations:** ^1^Stomatology Department, Centro Hospitalar Universitário de Lisboa Central, Lisbon, Portugal; ^2^Stomatology Department, Hospital de São Bernardo, Setúbal, Portugal

## Abstract

Brain abscess is a very rare condition but has a significant mortality rate. The three main routes of inoculation are trauma, contiguous focus, and the hematogenous route. The odontogenic focus is infrequent and is usually a diagnosis of exclusion. This paper presents a brain abscess case proven to be of dental origin, caused by *Actinomyces meyeri* and *Fusobacterium nucleatum*. This case highlights the risk underlying untreated dental disease and why oral infectious foci removal and good oral health are essential in primary care.

## 1. Introduction

Brain abscess is the most common type of focal infectious neurologic lesion and is defined as a localized area of suppuration which develops within the brain parenchyma [1–5] after inoculation with a pathogen [6].

In most literature, its prevalence is reported as 1 : 100.000 [1, 2, 4, 5, 7, 8], which usually occurs more frequently in men [5, 7, 8] under 60 years of age [5].

Even with modern antibiotic treatment, it is still considered a life-threatening infection [2–6, 8, 9] with a mortality rate ranging from 10% [2, 4, 8] to 24% [3, 6].

There are three inoculation pathways from which a brain abscess can develop [2–6, 9–13]: direct contamination through a surgical event (10%–20% of cases) [6, 12], hematogenous spread (20%–30% of cases) [12], and spread from a contiguous focus (20%–40% of cases) [6, 12].

Cerebral space lesion presents roughly the same signs and symptoms: headache (first and most common) [5, 8, 14–16] and nausea/vomiting [9] as a result of increased intracranial pressure [2, 8, 15, 16]. The poorer the mental status of the patient upon admission, the poorer the long-term outcome will be [9], defining this as a major prognosis indicator [8].

Odontogenic infections are a rare but known possible cause of brain abscesses [4, 5, 8, 13, 17, 18] via hematogenous spread [14]. Recent dental treatment, poor oral hygiene, and diabetes are also know risk factors for brain abscess development due to the transient bacteremia associated with compromised immunity [5, 14]. Periodontitis, defined as an infection of tooth-supporting tissues, is characterised by an inflamed and necrotic area with the destruction of the alveolar bone [19, 20]. This condition is an obvious starting point of bacteremia and metastatic spread [2, 5], due to the great heterogeneity and load of microorganisms. Other potential odontogenic sources are odontogenic cysts and periapical osteitis [4].

Comparison cultures from brain abscess and oral focus are rarely obtainable [8], thus making this an exclusion diagnosis [4, 8, 12, 18] with supportive evidence often limited to a positive culture for oral flora from drained cerebral suppuration [8, 12]. Negative cultures of abscesses may be due to cultivation failure or previous antibiotic treatment [21], and this can lead to controversy regarding the accurate prevalence of brain abscess of odontogenic origin, with literature numbers ranging from 3% [7] to 30% [12].

Brain abscesses are frequently polymicrobial [3, 5, 8, 11–13], and those with odontogenic origin have Streptococci as the primary isolate [2, 3, 5, 8, 12]. *Fusobacterium nucleatum* and *Actinomyces meyeri* are both rare isolates in this context [22, 23].

Among the family of Fusobacteriaceae [22, 24], *Fusobacterium nucleatum* are Gram-negative anaerobic rod-shaped bacilli [24–26] and the most pathological species of the genera *Fusobacterium* [22, 27]. They are present in mucomembraneous surfaces such as the oral cavity [28], respiratory, gastrointestinal, and genitourinary tracts [24, 26, 27] and are reported to cause periodontal disease [24, 26]. Their embolic characteristics [27] are known to cause 6% of all bacterial brain abscesses [24], which is a rare finding [22, 24, 26].

*Actinomyces meyeri* belongs to the family of Actinomycetaceae and is an anaerobic, branching-filamentous Gram-positive bacillus [17, 19, 28–34] present in the oral flora and gastrointestinal and urogenital tracts [15, 17, 28–30, 32, 34–36]. *Actinomyces meyeri* is usually found in the periodontal sulcus [19, 34, 37, 38], and while it is considered a low-virulence bacteria in immunocompetent individuals, there are risk factors identified for *Actinomyces* infection including poor oral hygiene, abusive alcohol intake, pulmonary infection, and recent dental treatment [17, 30, 34, 36, 38]. Its infection is usually restricted to the cervicofacial region, with the central nervous system rarely affected [15, 17, 28, 34, 36, 38–41], accounting for approximately 3% of actinomycosis [33, 35, 42]. Infection by *Actinomyces meyeri* itself is very rare [28, 30, 35], accounting for only 1% of all *Actinomyces* spp. involved in human pathogenesis [31, 39].

In 2017, a search was performed for case reports, case series, clinical trials, and reviews published in English in peer-reviewed journals in PubMed, using the MeSH terms actinomyces, actinomycosis, *Actinomyces meyeri*, brain abscess, cerebral, and/or central nervous system, by Guillamet et al. Only seven case reports were found for brain abscess by *Actinomyces meyeri*, and only one was also positive for *Fusobacterium nucleatum* [28, 29].

Early diagnosis with CT scan and appropriate treatment improves not only the mortality rate but the overall prognosis as well [3, 6–9, 16, 43].

The treatment for brain abscess is usually a combination of surgery and long-term antibiotics [2, 4, 8, 10, 12, 13, 20, 21, 23, 24, 28, 29, 31, 33, 37, 43] with oral cavity sanitisation when oral focus is suspected [3, 4, 12, 13].

The following case reports an immunocompromised patient with no recent history of dental treatment, but with several other risk factors for cerebral abscess, who developed a brain abscess. The culture was positive (before any antibiotic treatment) for *Fusobacterium nucleatum* and *Actinomyces meyeri*. He was submitted to cerebral drainage and long-term antibiotic treatment, having only mild left crural paresis and left temporal hemianopsia as sequelae.

## 2. Case Report

A 60-year-old man was transferred from the Centro Hospitalar do Médio Tejo-Hospital de Abrantes due to complaints of headache, barely perceptible speech, and decreased muscle strength in the left hemibody, with a week of onset, associated with partial seizures in the left hemibody. The comorbidities were non-insulin-treated type 2 diabetes mellitus, medicated glaucoma, treated gastric cancer, smoking, and daily alcohol abuse. The patient reported no regular dental follow-up, presenting only in urgent cases.

**Figure 1:**
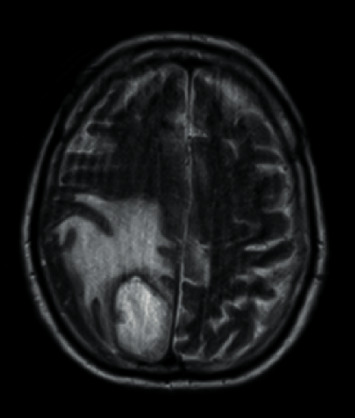
CE-CT in the latter hospital.

At the latter hospital, the Abdominopelvic Computed Tomography (AP-CT) revealed no significant changes and the Cranioencephalic Computed Tomography (CE-CT) revealed an “apparently intra-axial expansive lesion in the right parasagittal parieto-occipital area, predominantly hypodense, with about 4 cm in diameter, bordered by an extensive halo of perilesional edema” (Figure 1).

During admission, at the Emergency Department of Hospital de São José, the patient presented a Glasgow Score of 15, left hemibody myoclonus, grade 3 left hemiparesis, and left labial commissure deviation. No other changes in physical examination were sought. Laboratory analysis revealed erythropenia of 3.29 × 1012/L (normal: 4.4–5.9 × 1012/L); 34.9% hematocrit (normal: 40–50%); neutrophilia of 77.9% (normal: 40–75%); lymphopenia of 12.9% (normal: 15–45%); hyperglycemia of 143 mg/dL (normal: 60–100 mg/dL); uremia of 16 mg/dL (normal: 18–55 mg/dL); and hyponatraemia of 134 mEq/L (normal: 136–145 mEq/L).

**Figure 2:**
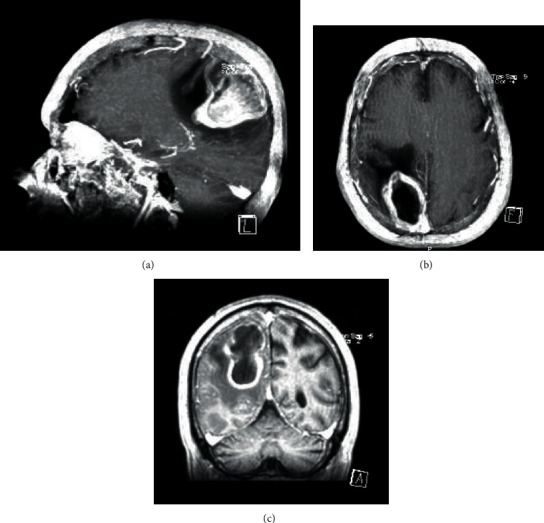
CE-MRI in Hospital São José.

Taking into consideration the findings of CE-CT, the most likely diagnoses were neoplasm or infection of the Central Nervous System (CNS), so the patient was transferred to the Neurosurgery Department. A cranioencephalic Magnetic Resonance Imaging (CE-MRI) was also performed, which revealed a “right posterior parietal corticosubcortical lesion, with diffusion restriction, annular enhancement after gadolinium, and extensive perilesional edema, being more in favor of an abscessed collection hypothesis” (Figures 2(a)–2(c)).

The patient was submitted to surgical drainage under general anesthesia, with the purulent content sent to microbiology analysis and initiation of empirical antibiotic therapy with ceftriaxone 2g every 12 hours and clindamycin 600 mg every 6 hours. The postoperative period in the Postanesthetic Intensive Care Unit (PICU) was uneventful, and the patient was transferred back to the Neurosurgery Department after 2 days.

At the microbiology department, the pus aspirate sample was cultured aerobically and anaerobically, the latter being performed through placing an inoculated medium into an anaerobic environment jar. The sample was initially placed in a liquid anaerobic medium (Brain Heart Infusion broth) enriched with peptone, glucose, sodium chloride, and disodium phosphate. Three days later, a colony growth measured by turbidity was verified. Then, a subculture was performed in different types of growth solid medium (blood agar, chocolate agar, MacConckey agar, and CHROMagar MRSA). A direct identification with matrix-assisted laser desorption/ionization time-of-light mass spectrometry (MALDI-TOF MS) from the anaerobic subculture colonies was performed, and the result was *Actinomyces meyeri* and *Fusobacterium nucleatum*. Due to cost-effectiveness issues, antimicrobial susceptibility testing of anaerobes is not carried out in our microbiology department, and the antibiotic therapy is established according to the published surveillance data of our hospital.

At this point, the stomatology evaluation and infectious diseases collaboration was requested, which concluded that the two microorganisms had oropharyngeal origin, and thus, an adjustment of antibiotic coverage was recommended. The patient started penicillin 24 MUI a day and metronidazole 500 mg every 8 hours, intravenously for 3 weeks and orally thereafter.

**Figure 3:**
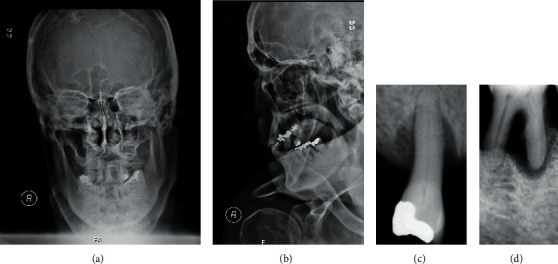
Face X-ray and intraoral X-ray.

The patient was observed at the Stomatology Department of Hospital de São José, where several oral septic foci were identified that could justify the necessary bacteremia for hematogenous inoculation. Oral physical examination identified partial edentulism in both dental arches; poor oral hygiene with the presence of bacterial plaque and calculus; and generalized advanced chronic periodontitis with active periodontal pockets (Figures 3(a)–3(d)). No oral culture was performed due to our experience with failure to achieve conclusive bacterial cultures under a polymicrobial oral flora with previous antibiotic treatments. The treatment plan consisted of systematic elimination of oral septic foci, teaching and motivation for oral hygiene, and the investigation of possible immune deficits. This evaluation showed no immunodeficiency.

**Figure 4:**
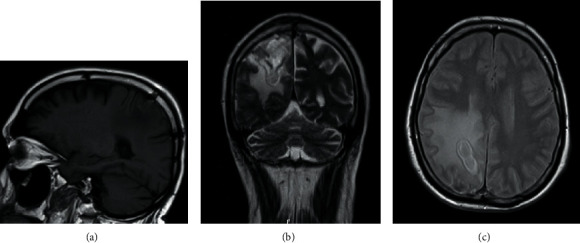
CE-MRI at 12 hospitalisation day.

**Figure 5:**
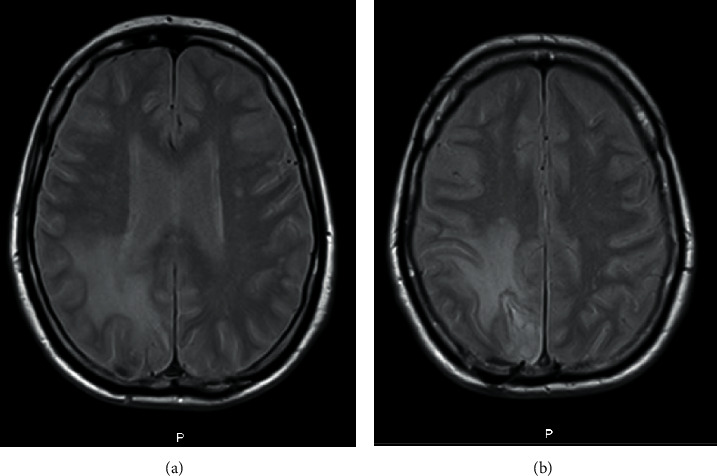
CE-MRI at 28 hospitalisation day.

During hospitalisation, the patient underwent two control Cranioencephalic Magnetic Resonance Imaging (CE-MRI) (the first one at 12 hospitalisation day (Figures 4(a)–4(c)) and the second one at 28 hospitalisation day (Figures 5(a) and 5(b))) which revealed downsizing of the abscessed collection and perilesional edema. After one month of hospitalisation, the patient was transferred to the residence area hospital, with the requirement to complete the antibiotic therapy cycle, perform physiotherapy treatments, and repeat the CE-MRI. The patient was left with only residual neurological deficits as a sequel.

## 3. Discussion

Brain abscess is a rare but serious condition [2, 3, 5, 7, 22, 26, 42]. The diagnosis is not always straightforward, but CT and MRI are a helpful tool for the clinicians [2, 3, 7, 17, 40]. Although there were no comparison cultures between the patient's oral flora and brain abscess, the fact that all other causes were excluded (including any recent trauma), combined with the fact that he had advanced, generalized chronic periodontitis and that both isolated species are found in normal oral flora, makes this a case of brain abscess of likely dental origin [13, 17, 19, 24, 26, 29, 31, 42].

The patient has residual neurological deficits and still has regular follow-up.

A literature search for case reports with brain abscess caused by *Actinomyces meyeri* and *Fusobacterium nucleatum* returned in very few results, and none were found with these two associated microorganisms [3, 29, 39, 40].

This case emphasises the underestimated risk associated with untreated dental disease. Good oral health and regular dental examinations are crucial in every patient, not only for the widely known reason but also to prevent metastatic infections which could follow any odontogenic chronic infection.

## Data Availability

No data were used to support this study.
